# Editorial: Research advances in understanding the etiology, epidemiology, pathophysiology, clinical features, and management of the Ehlers Danlos syndrome disorders

**DOI:** 10.3389/fmed.2024.1364308

**Published:** 2024-07-22

**Authors:** Clair A. Francomano, Anne Maitland, Deborah Krakow, Cheryl L. Maier

**Affiliations:** ^1^Department of Medical and Molecular Genetics, Indiana University School of Medicine, Indianapolis, IN, United States; ^2^Metrodora Institute, Salt Lake City, UT, United States; ^3^Department of Obstetrics and Gynecology, University of California, Los Angeles, Los Angeles, CA, United States; ^4^Department of Pathology and Laboratory Medicine, Emory University School of Medicine, Atlanta, GA, United States

**Keywords:** Ehlers-Danlos syndromes, Hypermobility Spectrum Disorders, pain, ocular manifestations, neuropsychiatric manifestations, quality of life, upper cervical instability

The Ehlers-Danlos syndromes (EDS) are a heterogeneous group of hereditable disorders of connective tissue, characterized primarily by joint hypermobility, skin hyperextensibility, and fragility of the tendons and ligaments. In 2013, Professor Rodney Grahame spoke at a colloquium sponsored by the Bobby Jones Chiari and Syringomyelia Foundation, and memorably proclaimed that “no other disease in the history of modern medicine has been neglected in such a way as the Ehlers-Danlos syndromes.” Over the ensuing decade, the biomedical community started to address that neglect. Of the 13 recognised types of EDS, the molecular basis is now known for twelve. The hypermobile type of EDS, which is the most common, is the only for which the molecular cause remains unknown. Work in several laboratories around the world is ongoing to identify the molecular cause of hypermobile EDS and biomarkers related to the condition. In 2016, experts from around the world gathered, in New York City, for the first meeting of the International Consortium on Ehlers-Danlos Syndromes and Hypermobility Spectrum Disorders. They came together to consider what was known about EDS from the vantage point of different medical specialties and the different types of EDS (hypermobile, classical, vascular, and rarer types). Each group was charged with documenting the state of knowledge within their specific area: what was known, what knowledge gaps existed, and what could be done to fill those gaps. New diagnostic criteria were developed for each EDS type and a framework for the diagnosis of joint hypermobility was put forward, allowing for introduction of the Hypermobility Spectrum Disorders (HSD), a spectrum of conditions with joint hypermobility which do not meet the diagnostic criteria for a recognized syndrome such as any of the types of EDS. Each group produced a manuscript for a dedicated issue of the American Journal of Medical Genetics, published in March 2017. The lead article in that issue, entitled “*The 2017 international classification of the Ehlers-Danlos syndromes*,” (1) has been cited nearly 1,000 times.

This Research Topic of eight articles for Frontiers in Medicine, entitled “*Research advances in understanding the etiology, epidemiology, pathophysiology, clinical features, and management of the ehlers-danlos syndrome disorders*”, extends our knowledge base on EDS and HSD. The Research Topic includes two qualitative studies, one on the array of associated pain syndromes and the second on the use of alternative and complementary medicine by persons living with hypermobile EDS. There are two review papers, one focusing on the extracutaneous features and complications of EDS and another one specifically on visual manifestations. A description of the neuroconnective endophenotype questionnaire and its validation discusses the interconnection of mind and body manifestations. The remaining articles, emerging from the allied health professions, include the following: balance and postural sway in patients with hypermobile EDS; biochemical changes in the gastrocnemius mediusAchilles tendon complex in people with HSD; and the presentation and management of upper cervical instability in people with symptomatic generalized joint hypermobility.

The qualitative study by Schubart et al. on pain and related symptoms supports the findings of Doyle and Halverson regarding the use of integrative medicine techniques among persons with EDS and HSD. Both papers recognize the high degree of resilience among this population, and highlight the need for increasing awareness among medical professionals regarding their clinical complexity, and the profound dearth of validated strategies for managing patients' concerns.

Doolan et al. performed a systematic review of the literature to ascertain the prevalence of extracutaneous features among the different types of EDS. In keeping with the findings of Doyle and Halverson and Schubart et al., pain was a common manifestation. Among the plethora of multisystem complications, ocular manifestations are noted, and these are expanded upon by the article in this Research Topic by Asanad et al.. Doolan et al. also found that neuropsychiatric complications have been reported in almost all types of EDS, a finding that endorses the importance of the work by Bulbena et al. in this Research Topic.

The crucial interaction between body and mind should come as no surprise to any student of human nature. It is important to emphasize that these findings do not imply that the experience of complex symptoms for people living with EDS and HSD is “all in their head,” as it has been pejoratively explained to many, but rather that the physiology of the body, impacted by the autonomic nervous system and the immune system, as well as the ubiquitous nature of the connective tissue, significantly perturb both physical and mental health.

The allied health professions, including physical therapists, are assuming an increasing role in the optimal management of persons with EDS and HSD. Balance and proprioception are often impaired in these disorders. This may contribute to a fear of movement (kinesiophobia) and reduced activity, exacerbating the musculoskeletal complications emerging from hypermobility. The paper by Whitmore et al. in this Research Topic presents their findings on balance and sway in a population of persons with hEDS, and offers a range of diagnostic tools to help assess proprioception and balance as a means of measuring therapeutic progress.

Alsiri and Palmer utilized sonoelastography to assess the biomechanical impact of HSD on the elasticity of the gastrocnemius medius-Achilles tendon (GM-AT) complex. Their finding of increased elasticity at the GM-AT complex is further evidence of underlying connective tissue involvement in the HSD phenotype.

Upper cervical instability, including instability at the craniocervical and/or atlo-axial junction, is likely under-recognized among persons with EDS and HSD. The work by Russek et al. presents expert consensus recommendations for screening, assessing, and managing the complex symptoms associated upper cervical instability in the setting of symptomatic generalized joint hypermobility. This is an important contribution to the literature on the topic, providing a guide for the diagnosis and conservative management of these potentially devastating complications.

Overall, EDS and HSD remain under-represented in the literature, but research efforts over the past decade have accelerated tremendously. The eight papers in this Research Topic of Frontiers in Medicine represent hope for the future of people living with EDS, HSD, and common co-morbidities, which so often dramatically impact quality of life. Much remains to be done.

## Author contributions

CF: Writing – original draft, Writing – review & editing. AM: Writing – review & editing. DK: Writing – review & editing. CM: Writing – review & editing.
